# Efficacy and safety of thrombopoietin receptor agonists in patients with primary immune thrombocytopenia: A systematic review and meta-analysis

**DOI:** 10.1038/srep39003

**Published:** 2016-12-19

**Authors:** Li Wang, Zhe Gao, Xiao-ping Chen, Hai-yan Zhang, Nan Yang, Fei-yan Wang, Li-xun Guan, Zhen-yang Gu, Sha-sha Zhao, Lan Luo, Hua-ping Wei, Chun-ji Gao

**Affiliations:** 1 Department of Hematology, Chinese People’s Liberation Army (PLA) General Hospital, Beijing, China; 2 Department of Hematology, Laoshan Branch, No. 401 Hospital of Chinese PLA, Qingdao, China; 3 Department of Hematology, The Fourth Hospital of Hebei Medical University, Shijiazhuang, China; 4 Department of Hematology, Navy General Hospital of Chinese PLA, Beijing, China; 5 Department of Hematology, Linyi People’s Hospital, Linyi, China

## Abstract

Immune thrombocytopenia (ITP) is an autoimmune disease characterized by increased platelet destruction and impaired platelet production. In this study, we conducted a systematic review and meta-analysis to determine the efficacy and safety of thrombopoietin receptor agonists (TPO-RAs) in primary ITP patients. Thirteen randomized controlled trials were included in this study, the pooled results of which demonstrated that TPO-RAs significantly increased platelet response (R) and durable response (DR) rates [risk ratio (RR): 2.77, 95% confidence interval (CI): 2.01–3.82, *P* = 5.9 × 10^−10^; RR: 7.52, 95% CI: 3.94–14.35, *P* = 9.2 × 10^−10^; respectively] and that TPO-RAs significantly reduced the incidences of any or severe bleeding events (RR: 0.80, 95% CI: 0.67–0.95, *P* = 0.013; RR: 0.52, 95% CI: 0.27–0.99, *P* = 0.048; respectively). Moreover, our results indicated that there was a significant reduction in the proportion of patients needing rescue medications in the TPO-RA groups compared with the control groups (RR: 0.50, 95% CI: 0.42–0.59, *P* = 2.0 × 10^−15^) and that the rates of any or severe adverse events were similar between the TPO-RA and control regimens (RR: 1.01, 95% CI: 0.92–1.10; RR: 0.74, 95% CI: 0.54–1.01; respectively). These findings demonstrate that TPO-RAs are an effective and safe second-line treatment option for primary ITP patients.

Primary immune thrombocytopenia (ITP), previously known as idiopathic thrombocytopenia purpura, is an autoimmune disease characterized by isolated thrombocytopenia occurring in the absence of any obvious causes or disorders that may cause thrombocytopenia^1^,^2^,^3^. Increased platelet destruction and impaired platelet production are both involved in the pathophysiology of ITP^4^,^5^,^6^,^7^,^8^. Thrombocytopenia occurs when platelet destruction exceeds platelet production^9^, and patients with persistently low platelet counts are often at a high risk for severe bleeding and mortality^10^. Therefore, the main goal of ITP therapy is to elevate platelet counts to a safe level to prevent severe bleeding and minimize the incidence of adverse events (AEs)^1^,^3^,^11^.

Traditional ITP treatment strategies, such as glucocorticosteroids, immunoglobulins, immunomodulatory agents, or splenectomy, primarily mitigate immune-mediated platelet destruction^12^,^13^,^14^. Although these strategies are usually effective, a number of patients are refractory to these therapies. Moreover, treatment-related side effects and treatment contraindications often limit the success and widespread use of the abovementioned strategies^10^,^15^,^16^,^17^,^18^. For example, splenectomy is a recommended second-line option for ITP patients; however, some patients relapse after splenectomy or even fail to respond to splenectomy^11^. Moreover, many patients are reluctant to undergo or have contraindications to this invasive procedure, and post-splenectomy complications, namely, the risk of sepsis, represent a deterrent to its routine performance^2^. Furthermore, a substantial number of ITP patients, namely, children, may remit spontaneously several months after diagnosis. Avoidance of splenectomy may benefit these patients^2^.

Thrombopoietin (TPO) is the main cytokine that stimulates thrombopoiesis, and although platelet counts are low in ITP patients, no compensatory increase in TPO production occurs in these patients^14^. Thrombopoietin receptor agonists (TPO-RAs) are TPO mimetics that can bind to and activate TPO receptors, leading to megakaryocyte maturation, proliferation and differentiation and resulting in increased platelet production^9^,^19^. Two major TPO-RAs, romiplostim and eltrombopag, have been investigated in several randomized controlled trials (RCTs) involving adult and pediatric ITP patients^9^,^12^,^13^,^14^,^15^,^16^,^19^,^20^,^21^,^22^,^23^,^24^,^25^, the results of which are encouraging. Currently, romiplostim and eltrombopag are recommended as second-line therapeutic options for adult ITP patients^2^,^3^.

However, several issues should be noted. First, the therapeutic effects of TPO-RAs vary greatly among relevant studies, and inconsistency exists with respect to the results of these studies. Second, the safety profiles of TPO-RAs are not completely reassuring, partly due to the relatively small sample sizes of the relevant studies. Third, the efficacy and safety of TPO-RAs in pediatric ITP patients have not been comprehensively reviewed. Thus, we conducted this systematic review and meta-analysis to comprehensively evaluate the efficacy and safety of TPO-RAs in adult and pediatric primary ITP patients.

## Results

### Study selection and characteristics

**Figure 1:**
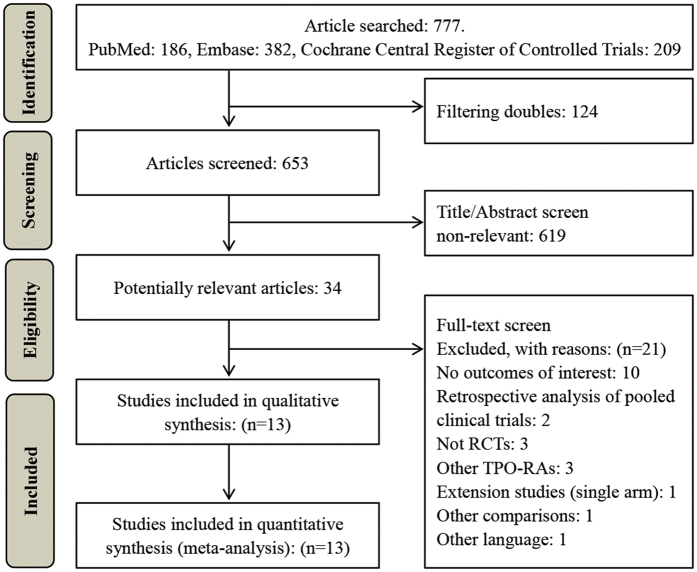
Study flow diagram. RCTs: randomized controlled trials; TPO-RAs: thrombopoietin receptor agonists.

A total of 777 articles were identified, and 653 articles remained after duplicates were removed. A total of 619 of these articles were removed after their titles and abstracts were screened. The full texts of 34 potentially relevant articles were subsequently screened, and 21 of these articles were excluded (the excluded articles and the reasons for their exclusion are listed in Supplementary Table S1). Finally, 13 studies involving 1,126 ITP patients were included in the systematic review and meta-analysis (Fig. 1).

The characteristics of the included studies are listed in Tables 1 and 2. All RCTs enrolled ITP patients with platelet counts of 30 × 10^9^/L or less, with the exception of one study that enrolled ITP patients with platelet counts of less than 50 × 10^9^/L 21. All the included patients were classified as having persistent or chronic ITP, according to the new ITP classification system^1^. Patients were also classified as failed or relapsed following more than one previous ITP treatment. Patients were allowed to receive concomitant ITP drugs, primarily corticosteroids, as long as the doses of these drugs had been stable for one month or longer before the start of the study. All studies excluded patients who had histories of bone marrow disorders or who had recently suffered arterial or venous thrombotic events.

The sample sizes of the included RCTs ranged from 18 to 234. Four studies evaluated the efficacy and safety of romiplostim in adults^9^,^15^,^20^,^21^, and three studies evaluated the efficacy and safety of the same treatment in children^16^,^22^,^25^. Six studies compared romiplostim with placebo^9^,^15^,^16^,^20^,^22^,^25^, and one study compared romiplostim with the standard of care for ITP, based on institutional practices or therapeutic guidelines^21^. Four studies regarding eltrombopag were conducted in adults^12^,^13^,^14^,^24^, and two studies regarding eltrombopag were conducted in children^19^,^23^. All six of these studies compared eltrombopag with placebo^12^,^13^,^14^,^19^,^23^,^24^. The durations of romiplostim and eltrombopag treatment ranged from 6 to 52 weeks and 6 to 26 weeks, respectively.

### Risk of bias

Randomization was mentioned in all of the included studies, and a detailed description of random sequence generation was provided in eight studies^12^,^13^,^14^,^15^,^16^,^19^,^20^,^23^. Allocation concealment was conducted adequately in these eight studies^12^,^13^,^14^,^15^,^16^,^19^,^20^,^23^. One study was reported as an open-label RCT^21^, and one study was reported as a single-blinded RCT^22^. The remaining 11 RCTs were reported as double-blinded RCTs^9^,^12^,^13^,^14^,^15^,^16^,^19^,^20^,^23^,^24^,^25^. All studies were considered to have a “low risk of bias” for items including “incomplete data” and “selective outcomes reporting”. All seven studies were sponsored by Amgen Inc, with the exception of one romiplostim trial, and all eltrombopag trials were sponsored by GlaxoSmithKline.

### Primary outcomes

#### Platelet response and durable response

**Figure 2:**
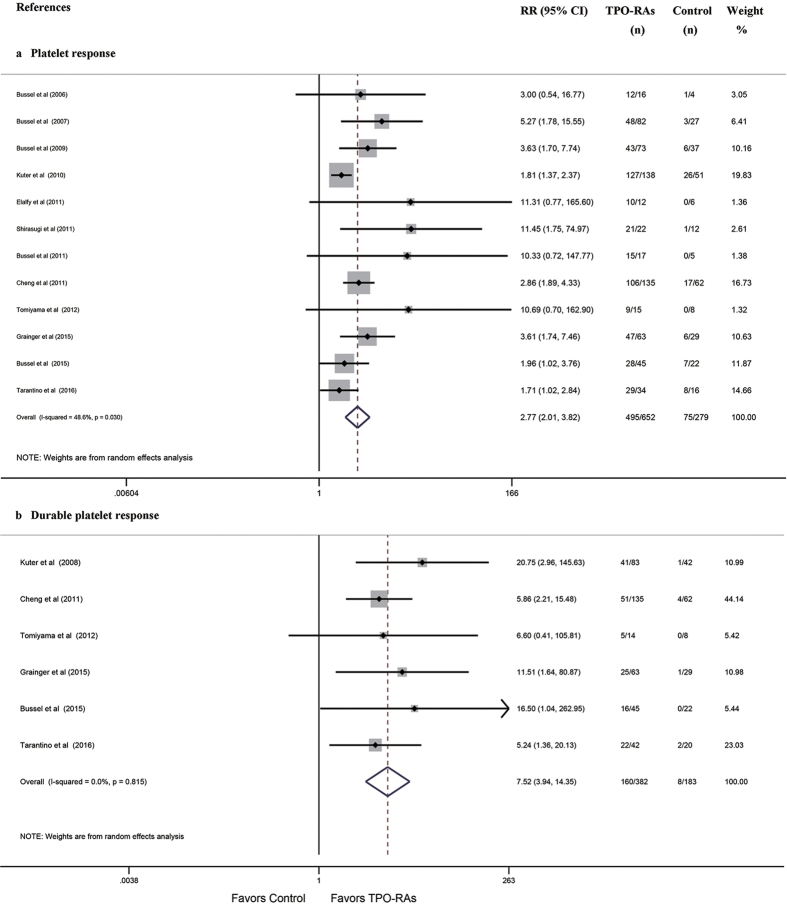
Forest plot and meta-analysis of the rates of R and DR. (**a**) R. (**b**) DR. TPO-RAs: thrombopoietin receptor agonists; RR: risk ratio; CI: confidence interval.

All 13 RCTs reported platelet response (R) or durable response (DR) rates. The pooled results demonstrated that TPO-RAs significantly increased the R (RR: 2.77, 95% CI: 2.01–3.82, *P* = 5.9 × 10^−10^ Fig. 2a) and DR rates (RR: 7.52, 95% CI: 3.94–14.35, *P* = 9.2 × 10^−10^ Fig. 2b). Subgroup meta-analysis based on TPO-RA regimens demonstrated that both romiplostim and eltrombopag were associated with higher rates of R (RR: 2.43, 95% CI: 1.40–4.22; RR: 3.01, 95% CI: 2.28–3.99; respectively) and DR (RR: 8.83, 95% CI: 2.19–35.61; RR: 7.21, 95% CI: 3.25–15.96; respectively Supplementary Fig. S1, Table 3), and subgroup meta-analysis based on different patient populations demonstrated that TPO-RAs substantially increased the rates of R or DR in children (RR: 2.49, 95% CI: 1.46–4.23; RR: 7.64, 95% CI: 2.73–21.36; for R and DR; respectively Supplementary Fig. S2, Table 3) and adults (RR: 3.13, 95% CI: 1.96–4.99; RR: 7.45, 95% CI: 3.25–17.08; for R and DR; respectively Supplementary Fig. S2, Table 3). Four studies reported the rates of R or DR for splenectomized vs. non-splenectomized ITP patients. The pooled results demonstrated that the rate of R was similar between splenectomized and non-splenectomized patients receiving TPO-RAs (RR: 0.84, 95% CI: 0.49–1.42 Supplementary Fig. S3). However, the rate of DR was significantly lower in splenectomized patients than in non-splenectomized patients receiving TPO-RAs (RR: 0.72, 95% CI: 0.54–0.95, *P* = 0.022 Supplementary Fig. S3).

#### Any or severe bleeding events

**Figure 3:**
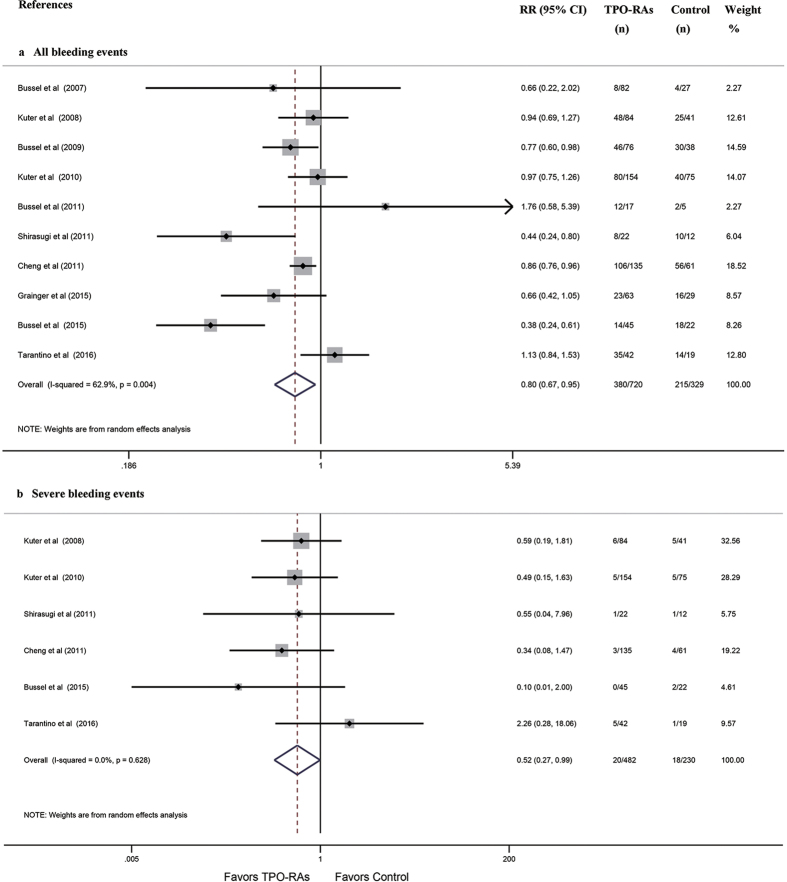
Forest plot and meta-analysis of the incidences of any and severe bleeding events. (**a**) any bleeding events. (**b**) severe bleeding events. TPO-RAs: thrombopoietin receptor agonists; RR: risk ratio; CI: confidence interval.

Ten of the included studies reported the incidence of any or severe bleeding events. The pooled results demonstrated that TPO-RAs significantly reduced the incidence of any or severe bleeding events (RR: 0.80, 95% CI: 0.67–0.95, *P* = 0.013; RR: 0.52, 95% CI: 0.27–0.99, *P* = 0.048; respectively Fig. 3a,b). Subgroup analysis based on TPO-RA regimens indicated that romiplostim did not substantially decrease the incidence of any or severe bleeding events (RR: 0.93, 95% CI: 0.72–1.21; RR: 0.64, 95% CI: 0.31–1.35; respectively Supplementary Fig. S4, Table 3). However, regarding eltrombopag, there were substantial reductions in the incidence of any or severe bleeding events in treated patients compared with control patients (RR: 0.68, 95% CI: 0.51–0.90; RR: 0.27, 95% CI: 0.07–1.00; respectively Supplementary Fig. S4, Table 3). Subgroup analysis based on different patient populations indicated that the incidence of any or severe bleeding events did not significantly decrease with TPO-RAs vs. placebo in pediatric ITP patients (RR: 0.78, 95% CI: 0.43–1.42; RR: 0.58, 95% CI: 0.03–12.09; respectively Supplementary Fig. S5, Table 3). However, the results of these studies indicated that there were considerable reductions in the incidence of any or severe bleeding events in adult ITP patients receiving TPO-RAs compared with control patients (RR: 0.84, 95% CI: 0.74–0.96; RR: 0.49, 95% CI: 0.24–0.97; respectively Supplementary Fig. S5, Table 3).

#### Secondary outcomes

**Figure 4:**
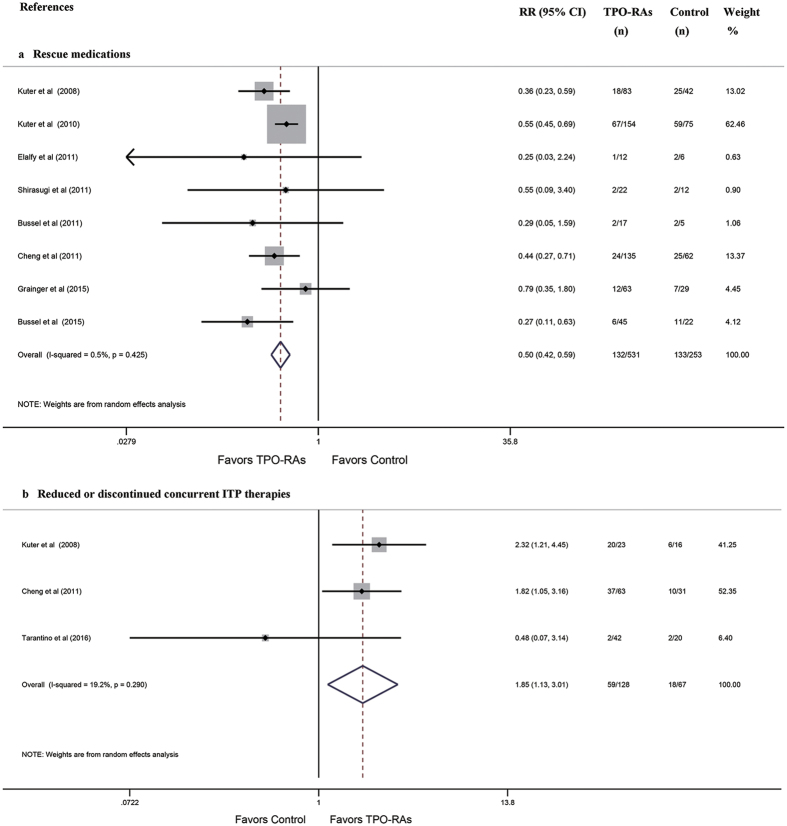
Forest plot and meta-analysis of the need for rescue medications and the numbers of patients who were able to reduce or discontinue concurrent ITP therapies. (**a**) rescue medications. (**b**) reduced or discontinued concurrent ITP therapies. ITP: immune thrombocytopenia; TPO-RAs: thrombopoietin receptor agonists; RR: risk ratio; CI: confidence interval.

The pooled results based on eight studies indicated that there was a significant reduction in the proportion of patients needing rescue medications in the TPO-RA groups compared with the control groups (RR: 0.50, 95% CI: 0.42–0.59, *P* = 2.0 × 10^−15^ Fig. 4a). Moreover, there was no substantial difference in the proportions of patients needing rescue medications between the different TPO-RA regimen or patient population subgroups (Supplementary Fig. S6, Table 3). In addition, the pooled results based on three studies indicated that there was a significant increase in the proportion of patients who were able to reduce or discontinue their concurrent ITP therapies in the TPO-RA group compared with the control group (RR: 1.85, 95% CI: 1.13–3.01, *P* = 0.014 Fig. 4b).

#### Safety profiles

**Figure 5:**
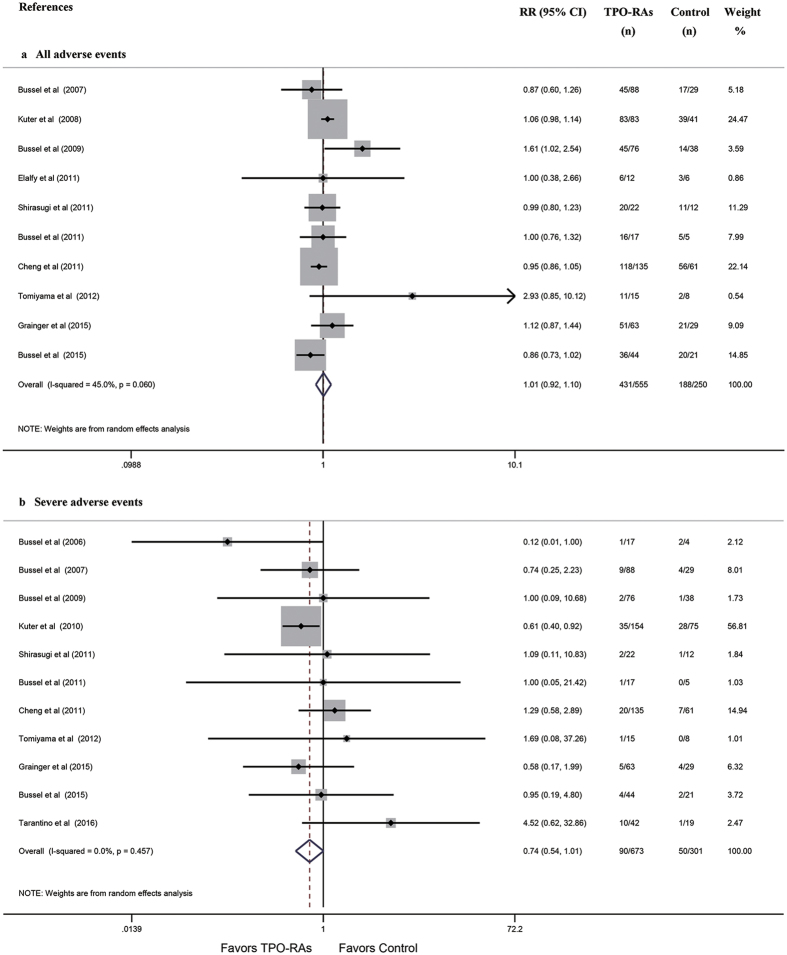
Forest plot and meta-analysis of the incidences of any and severe AEs. (**a**) any AEs. (**b**) severe AEs. TPO-RAs: thrombopoietin receptor agonists; RR: risk ratio; CI: confidence interval.

Thirteen studies reported the incidence of any or severe AEs. Based on data from 10 studies, the rates of any AEs were similar between the TPO-RA and control regimens (RR: 1.01, 95% CI: 0.92–1.10, *P* = 0.913 Fig. 5a). Additionally, based on data from 11 studies, the rates of severe AEs tended to be lower in the TPO-RA groups than in the control groups (RR: 0.74, 95% CI: 0.54–1.01, *P* = 0.054 Fig. 5b). No significant differences in the rates of AEs were found between the pre-specified subgroups (Supplementary Figs S7–8, Table 3).

The AEs of interest for TPO-RAs included thrombosis, bone marrow reticulin increases, and the generation of neutralizing antibodies to TPO-RAs or endogenous TPO^17^,^26^. There was no substantial difference in thrombotic events between the TPO-RA and control regimens (RR: 1.08; 95% CI: 0.40–2.93 Supplementary Fig. S9, Table 3). Neutralizing antibodies to either romiplostim or TPO were not detected in six studies^9^,^15^,^16^,^20^,^21^,^25^, and no bone marrow reticulin/fibrosis was detected in two studies^20^,^22^. One patient with an increased baseline bone marrow reticulin level exhibited an increased reticulin level during romiplostim treatment, but this level subsequently returned to baseline^15^. One patient receiving romiplostim exhibited an increased bone marrow reticulin level during a 6-month post-treatment follow-up period^21^, and two patients exhibited reversible increases in their bone marrow reticulin levels during the subsequent extension study^9^.

The AEs of interest for eltrombopag included hepatobiliary function abnormalities and increases in the incidence of cataracts^27^. There was a trend toward an increased incidence of liver function abnormalities with eltrombopag vs. placebo (RR: 2.13, 95% CI: 0.74–6.17 Supplementary Fig. S10, Table 3). There was no considerable difference between the eltrombopag and control regimens with respect to the incidence of cataracts (RR: 0.89, 95% CI: 0.42–1.91 Supplementary Fig. S10, Table 3).

## Discussion

Our systematic review and meta-analysis has several advantages over the previous meta-analysis conducted in 2011^28^. First, only six RCTs were included in the meta-analysis by Zeng *et al*., whereas 13 RCTs were included in our study. Therefore, the results of our meta-analysis may be more reliable and comprehensive than the results of the meta-analysis by Zeng *et al*. For example, the results of the previous meta-analysis, based on data from three RCTs, indicated that there was no significant difference in the incidence of severe bleeding events between the TPO-RA and control groups^28^, whereas the pooled results of our meta-analysis, based on the results of six RCTs, indicated that TPO-RAs significantly decreased the incidence of severe bleeding events in treated patients compared with control subjects. Second, compared with the previous meta-analysis, our analysis included more endpoints, such as the need for rescue medications and the numbers of patients who were able to reduce or discontinue concurrent ITP therapies. Third, we conducted subgroup meta-analysis based on different TPO-RA regimens (romiplostim vs. eltrombopag) or patient populations (adults vs. children) because our study included an adequate number of studies for performing such analyses.

Our meta-analysis results indicated that TPO-RAs significantly increased the rates of R or DR and reduced the incidences of any or severe bleeding events in persistent and chronic primary ITP patients compared with control subjects. Moreover, our results indicated that TPO-RAs significantly decreased the need for rescue medications and increased the numbers of patients who were able to reduce or discontinue concurrent ITP therapies. The incidence of AEs in the TPO-RA-treated groups was similar to that in the placebo groups, and there was a decreasing trend in the incidence of severe AEs in ITP patients receiving TPO-RAs compared with control subjects.

For persistent and chronic ITP patients, reducing the risk of severe bleeding may be more important than achieving specific platelet counts^12^. Our subgroup meta-analysis revealed that romiplostim did not significantly improve the incidence of any or severe bleeding events, which may be due to the fact that the effects of TPO-RA on bleeding events are affected by rescue medication use^16^,^29^. Patients in the TPO-RA groups needed less rescue medications than patients in the control group, which may have confounded our results regarding the incidence of bleeding events. Thus, using a composite bleeding episode endpoint including both bleeding events and/or rescue medication use or using duration-adjusted bleeding event rates may yield more detailed and accurate results than using separate endpoints^16^,^29^. For example, there was no significant difference in the incidence of any bleeding events between the romiplostim and placebo groups (83% vs. 74%), whereas the duration-adjusted rate of any bleeding events or composite bleeding episodes was significantly lower with romiplostim than with placebo in the study by Tarantino *et al*.^16^. Moreover, the incidence of bleeding events in the romiplostim group was higher than that in the placebo group (71% vs. 40%), whereas the duration-adjusted bleeding event rates were 7.3 and 11.9 in the romiplostim and placebo groups, respectively, in the study by Bussel *et al*.^25^. Thus, the insignificance of the effects of TPO-RAs in pediatric ITP patients was subsequently attributed to these two studies^16^,^25^.

It has been noted that platelet responses following TPO-RA treatment commonly gradually decrease once medications are stopped, which has raised questions regarding whether the combination of TPO-RAs with other drugs can exert additive effects and provide better clinical benefits than TPO-RAs alone. Two RCTs conducted in China compared the concomitant use of recombinant human thrombopoietin (rhTPO) with rituximab or danazol monotherapy^30^,^31^, the results of which indicated that concomitant rhTPO use significantly increased the rate of R and shortened the time needed to achieve this response compared with rituximab or danazol monotherapy^30^,^31^. Moreover, the combination of rhTPO with rituximab was associated with an increased sustained response compared with rituximab monotherapy^30^. These two studies were not included in this study due to the presence of significant heterogeneity with respect to patient characteristics and therapy schedules. Moreover, the efficacy of rhTPO monotherapy, the efficacy and safety of rhTPO in Western ITP patients, and the efficacy of the combination of romiplostim or eltrombopag with other drugs for ITP require further clarification in future studies.

Several issues should be noted. First, the pooled results of this analysis must be interpreted with caution due to the heterogeneity of the included studies. All persistent, chronic and refractory adult and pediatric ITP patients were included in the analysis. Patient characteristics (age, gender, ITP duration, splenectomy status), TPO-RA regimens (TPO-RA types, starting doses, dosing algorithms, and treatment durations), and outcome of interest definitions varied among the included studies. Second, close monitoring of hepatobiliary laboratory test results is recommended during eltrombopag treatment due to an increasing trend in the incidence of liver function abnormalities among ITP patients receiving this treatment. Third, worsening thrombocytopenia may occur after TPO-RA discontinuation^9^. Therefore, close monitoring of platelet counts and bleeding risks is recommended after TPO-RA discontinuation^3^,^32^. Finally, whether the youngest patients have lower response rates to TPO-RAs than older patients needs to be clarified further in future studies^16^,^23^.

In conclusion, this systematic review and meta-analysis demonstrated that TPO-RAs are effective and safe agents for the treatment of primary ITP, and the results of this analysis support the use of TPO-RAs as second-line treatments for persistent and chronic ITP patients who are unresponsive to or relapse after receiving previous first-line ITP therapies.

## Methods

### Search strategy

We conducted a systematic literature search of PubMed, the Excerpta Medica Database (Embase), and the Cochrane Central Register of Controlled Trials (CENTRAL) to identify RCTs evaluating the efficacy of TPO-RAs in patients with primary ITP. We searched for trials published until May 2016 and restricted our search to English language studies. We used the following combination of search terms: “thrombocytopeni*”, “ITP”, “romiplostim”, “eltrombopag”, and “thrombopoietin”. Our detailed search criteria are listed in Supplementary Tables S2–4. We searched the references of the identified studies manually to find additional studies.

### Selection criteria

All RCTs involving patients with a confirmed diagnosis of primary ITP that compared TPO-RAs to placebo were considered, as were RCTs that included TPO-RAs as an additive to other drugs/therapies, as long as the additional drugs/therapies were identical between the two groups. RCTs reporting at least one outcome of interest were included in the analysis.

The primary outcomes of interest included the rates of R or DR and the incidences of any or severe bleeding events. R was defined as achieving platelet counts of more than 50 × 10^9^/L at any time during TPO-RA treatment, and DR was defined as maintaining R for ≥60% of the duration of TPO-RA treatment or maintaining R for six or more weeks during the final eight weeks of TPO-RA treatment. Bleeding severity was assessed according to the National Cancer Institute Common Terminology Criteria for Adverse Events (CTCAE), the World Health Organization (WHO) bleeding scale, or a grading scale specified for pediatric ITP patients^33^. Severe bleeding events were defined as bleeding events ≥ grade 3, according to the National Cancer Institute CTCAE and the WHO bleeding scale, or bleeding events ≥ grade 4, according to the criteria developed by Buchanan and Adix^33^.

The secondary endpoints of interest included the need for rescue medications and the numbers of patients who were able to reduce or discontinue concomitant ITP therapies. Rescue medications were defined as any new drugs or as increased doses or frequencies of concomitant drugs that were administered for the treatment of ITP, as well as platelet transfusions or splenectomies that were performed during TPO-RA treatment.

Safety profiles were assessed according to the incidence of AEs, which were graded according to the National Cancer Institute CTCAE.

### Data extraction and methodological quality evaluation

Data extraction was performed by two independent researchers. All related data, including reference details (the first author, publication year, and study design), participant characteristics (age, gender, ITP duration, splenectomy status, and sample size), TPO-RA regimens, the abovementioned primary and secondary endpoints, and safety profiles, were extracted. All discrepancies were resolved by discussion or consultation with a specialist. The corresponding authors of the included studies were contacted if necessary.

The methodological quality of the included studies was assessed according to the following seven items: adequacy of randomization, allocation concealment, blinding of participants, outcome assessors, incomplete data, selective reporting, and other biases, according to the Cochrane Collaboration Reviewers’ Handbook (Version 5.1.0)^34^.

### Statistical analysis

All statistical analyses were performed using Stata software (version 12.0, Stata Corporation, College Station, TX, USA). Risk ratios (RRs) and 95% confidence intervals (CIs) were used to pool binary outcomes. Heterogeneity between studies was quantified using the *I*^2^ statistic, and *I*^2^ > 50% and *P* < 0.10 indicated the presence of significant heterogeneity between studies^35^. Meta-analyses were conducted using random effects models, irrespective of whether heterogeneity existed or not. Pre-specified subgroup meta-analysis based on the different TPO-RA regimens (romiplostim vs. eltrombopag) and patient populations (adults vs. children) of the included studies were also conducted. *P* < 0.05 was considered statistically significant.

## Additional Information

**How to cite this article**: Wang, L. *et al*. Efficacy and safety of thrombopoietin receptor agonists in patients with primary immune thrombocytopenia: A systematic review and meta-analysis. *Sci. Rep.* **6**, 39003; doi: 10.1038/srep39003 (2016).

**Publisher's note:** Springer Nature remains neutral with regard to jurisdictional claims in published maps and institutional affiliations.

## Supplementary Material

Supplementary Information

## Figures and Tables

**Table 1:** Patient characteristics for the included randomized controlled trials.

Reference	Sample size (n)	Age (years)		Gender: F/M (n)		Duration of ITP (years)		Splenectomy status (yes/no) (n)	
		TPO-RA	Control	TPO-RA	Control	TPO-RA	Control	TPO-RA	Control
Romiplostim									
Bussel *et al*.^9^	21	45 (20–63); 53 (19–62)	55 (39–64)	12/5	3/1	5.6 (0.5–24.9); 9.1 (0.4–37.0)	3.4 (0.8–3.7)	13/4	1/3
Kuter *et al*.^15^	63	51 (27–88)	56 (26–72)	27/15	11/10	7.8 (0.6–44.8)	8.5 (1.1–31.4)	42/0	21/0
Kuter *et al*.^15^	62	52 (21–80)	46 (23–88)	27/14	16/5	2.2 (0.1–31.6)	1.6 (0.1–16.2)	0/41	0/21
Kuter *et al*.^21^	234	58 (18–90)	57 (18–86)	85/72	46/31	2.1 (0.0–44.2)	2.3 (0.0–33.2)	0/157	0/77
Shirasugi *et al*.^20^	34	58.5 ± 12.6	47.6 ± 13.4	14/8	10/2	9.7 (10.4)	7.6 (5.9)	10/12	5/7
Bussel *et al*.^25^	22	9 (1–17)	11 (2–14)	4/13	2/3	2.4 (0.8–14.0)	4.1 (0.6–8.6)	6/11	2/3
Elalfy *et al*.^22^	18	9.5 (2.5–16)	7 (4–15)	2/10	3/3	2.3 (1.2–7.0)	3.0 (1.5–6.5)	0/12	0/6
Tarantino *et al*.^16^	62	10 (6–14)	7.5 (6.5–13.5)	24/18	11/9	1.9 (1.0–4.2)	2.2 (1.5–3.7)	1/41	1/19
Eltrombopag									
Bussel *et al*.^24^	117	51 (23–79); 45 (23–81); 55 (18–85)	42 (18–85)	57/31	16/13	>0.5	>0.5	41/47	14/15
Bussel *et al*.^14^	114	47 (19–84)	51 (21–79)	43/33	27/11	>0.5	>0.5	31/45	14/24
Cheng *et al*.^13^	197	47.0 (34–56)	52.5 (43–63)	93/42	43/19	>0.5	>0.5	50/85	21/41
Tomiyama *et al*.^12^	23	58.0 (26–72)	60.5 (38–72)	8/7	7/1	>0.5	>0.5	11/4	5/3
Bussel *et al*.^23^	67	9 (8–10)	10 (8–12)	27/18	13/9	>0.5	>0.5	5/40	0/22
Grainger *et al*.^19^	92	9.4 (8.2–10.5)	9.8 (8.3–11.3)	30/33	14/15	3.4 (2.8)	4.4 (3.4)	4/59	0/29

Note: Data for age and the duration of ITP are shown as the median (range), median (interquartile range) or mean (standard deviation). Abbreviation: TPO-RAs: thrombopoietin receptor agonists; ITP: immune thrombocytopenia; F: female; M: male.

**Table 2:** Summary of the characteristics of the included randomized controlled trials.

Reference	Study design	TPO-RA regimens	Outcomes of interest
Romiplostim			
Bussel *et al*.^9^	Multicenter, double-blind, RCT	1 or 3 μg/kg SC weekly for 6 weeks, no dose adjustments.	Efficacy: R. Safety: AEs.
Kuter *et al*.^15^	Multicenter, double-blind, RCT	Starting dose of 1 μg/kg SC weekly for 24 weeks; dose was adjusted to achieve target platelet counts of 50 to 200 × 10^9^/L.	Efficacy: DR, bleeding events, rescue medications, concurrent therapies. Safety: AEs.
Kuter *et al*.^21^	Multicenter, open-label, RCT	Starting dose of 3 μg/kg SC weekly for 52 weeks; dose was adjusted to achieve target platelet counts of 50 to 200 × 10^9^/L.	Efficacy: R, bleeding events, rescue medications. Safety: AEs.
Shirasugi *et al*.^20^	Multicenter, double-blind, RCT	Japanese patients: starting dose of 3 μg/kg SC weekly for 12 weeks; dose was adjusted to achieve target platelet counts of 50 to 200 × 10^9^/L.	Efficacy: R; bleeding events, rescue medications. Safety: AEs.
Bussel *et al*.^25^	Multicenter, double-blind, RCT	Starting dose of 1 μg/kg SC weekly for 12 weeks; dose was adjusted to achieve target platelet counts of 50 to 250 × 10^9^/L.	Efficacy: R, bleeding events, rescue medications. Safety: AEs.
Elalfy *et al*.^22^	Single-center, single-blind, RCT	Starting dose of 1 μg/kg SC weekly for 12 weeks; doses were escalated to 5 μg/kg and then tapered.	Efficacy: R, rescue medications. Safety: AEs.
Tarantino *et al*.^16^	Multicenter, double-blind, RCT	Starting dose of 1 μg/kg SC weekly for 24 weeks; dose was adjusted to achieve target platelet counts of 50 to 200 × 10^9^/L.	Efficacy: R, DR, bleeding events, concurrent therapies. Safety: AEs.
Eltrombopag			
Bussel *et al*.^24^	Multicenter, double-blind, RCT	30, 50, or 75 mg/d orally for 6 weeks.	Efficacy: R, bleeding events. Safety: AEs.
Bussel *et al*.^14^	Multicenter, double-blind, RCT	50 mg orally daily for 6 weeks; dose was adjusted based on platelet counts.	Efficacy: R, bleeding events. Safety: AEs.
Cheng *et al*.^13^	Multicenter, double-blind, RCT	50 mg orally daily for 6 months; dose was adjusted based on platelet counts.	Efficacy: R, DR, bleeding events, rescue medications, concurrent therapies. Safety: AEs.
Tomiyama *et al*.^12^	Multicenter, double-blind, RCT	Japanese patients: starting dose of 12.5 mg (maximum dose of 50 mg) orally daily for 6 weeks; dose was adjusted based on platelet counts.	Efficacy: R, DR. Safety: AEs.
Bussel *et al*.^23^	Multicenter, double-blind, RCT	Starting doses of 37.5 mg/d orally daily for 7 weeks for patients aged 12–17 years, 50 mg/d orally daily for 7 weeks for patients aged 6–11 years weighing 27 kg or more, 25 mg/d orally daily for 7 weeks for patients aged 6–11 years weighing less than 27 kg, and 1.5 mg/kg/d orally daily for 7 weeks for patients aged 1–5 years (east Asian patients received half-doses); doses were adjusted to achieve target platelet counts of 50 to 200 × 10^9^/L.	Efficacy: R, DR, bleeding events, rescue medications. Safety: AEs.
Grainger *et al*.^19^	Multicenter, double-blind, RCT	Starting doses of 25–50 mg/d orally daily for 13 weeks for patients aged 6–17 years and 0.8–1.2 mg/kg/d orally daily for 13 weeks for patients aged 1–5 years; doses were adjusted based on platelet counts.	Efficacy: R, DR, bleeding events, rescue medications. Safety: AEs.

Abbreviation: TPO-RA: thrombopoietin receptor agonists; RCT: randomized controlled trial; SC: subcutaneously; R: platelet response; DR: durable platelet response; AEs: adverse events.

**Table 3:** Summary of the pooled results regarding the efficacy and safety of TPO-RAs in ITP patients.

Outcomes	Pooled RR (95% CI)	Romiplostim vs. eltrombopag			Children vs. adults		
		TPO-RA regimen	No. of studies	RR (95% CI)	Patient population	No. of studies	RR (95% CI)
Platelet response	2.77 (2.01–3.82)	Romiplostim	6	2.43 (1.40–4.22)	Children	5	2.49 (1.46–4.23)
		Eltrombopag	6	3.01 (2.28–3.99)	Adults	7	3.13 (1.96–4.99)
Durable platelet response	7.52 (3.94–14.35)	Romiplostim	2	8.83 (2.19–35.61)	Children	3	7.64 (2.73–21.36)
		Eltrombopag	4	7.21 (3.25–15.96)	Adults	3	7.45 (3.25–17.08)
Any bleeding events	0.80 (0.67–0.95)	Romiplostim	5	0.93 (0.72–1.21)	Children	4	0.78 (0.43–1.42)
		Eltrombopag	5	0.68 (0.51–0.90)	Adults	6	0.84 (0.74–0.96)
Severe bleeding events	0.52 (0.27–0.99)	Romiplostim	4	0.64 (0.31–1.35)	Children	2	0.58 (0.03–12.09)
		Eltrombopag	2	0.27 (0.07–1.00)	Adults	4	0.49 (0.24–0.97)
Rescue medications	0.50 (0.42–0.59)	Romiplostim	5	0.51 (0.42–0.62)	Children	4	0.42 (0.22–0.79)
		Eltrombopag	3	0.45 (0.27–0.75)	Adults	4	0.50 (0.42–0.60)
Reduce or discontinue concurrent ITP therapies	1.85 (1.13–3.01)						
Any adverse events	1.01 (0.92–1.10)	Romiplostim	4	1.05 (0.97–1.12)	Children	4	0.96 (0.83–1.11)
		Eltrombopag	6	1.03 (0.85–1.25)	Adults	6	1.03 (0.91–1.17)
Severe adverse events	0.74 (0.54–1.01)	Romiplostim	5	0.75 (0.28–2.03)	Children	4	1.03 (0.42–2.50)
		Eltrombopag	6	0.95 (0.57–1.60)	Adults	7	0.70 (0.50–0.98)
Thrombotic events	1.08 (0.40–2.93)						
Liver function abnormalities (eltrombopag)	2.13 (0.74–6.17)						
Cataracts (eltrombopag)	0.89 (0.42–1.91)						

Abbreviation: TPO-RAs: thrombopoietin receptor agonists; ITP: immune thrombocytopenia; RR: risk ratio; CI: confidence interval.

## References

[b1] Rodeghiero F. . Standardization of terminology, definitions and outcome criteria in immune thrombocytopenic purpura of adults and children: report from an international working group. Blood 113, 2386–2393 (2009). 19005182 10.1182/blood-2008-07-162503

[b2] Provan D. . International consensus report on the investigation and management of primary immune thrombocytopenia. Blood 115, 168–186 (2010). 19846889 10.1182/blood-2009-06-225565

[b3] Neunert C. . The American Society of Hematology 2011 evidence-based practice guideline for immune thrombocytopenia. Blood 117, 4190–4207 (2011). 21325604 10.1182/blood-2010-08-302984

[b4] Ballem P. J. . Mechanisms of thrombocytopenia in chronic autoimmune thrombocytopenic purpura. Evidence of both impaired platelet production and increased platelet clearance. J. Clin. Invest. 80, 33–40 (1987). 3597777 10.1172/JCI113060 PMC442198

[b5] Olsson B. . T-cell-mediated cytotoxicity toward platelets in chronic idiopathic thrombocytopenic purpura. Nat. Med. 9, 1123–1124 (2003). 12937414 10.1038/nm921

[b6] Zhang F. . Cell-mediated lysis of autologous platelets in chronic idiopathic thrombocytopenic purpura. *Eur*. J. Haematol. 76, 427–431 (2006). 10.1111/j.1600-0609.2005.00622.x 16480433

[b7] Chang M. . Immune thrombocytopenic purpura (ITP) plasma and purified ITP monoclonal autoantibodies inhibit megakaryocytopoiesis *in vitro*. Blood 102, 887–895 (2003). 12676790 10.1182/blood-2002-05-1475

[b8] McMillan R., Wang L., Tomer A., Nichol J. & Pistillo J. Suppression of *in vitro* megakaryocyte production by antiplatelet autoantibodies from adult patients with chronic ITP. Blood 103, 1364–1369 (2004). 10.1182/blood-2003-08-2672 14576051

[b9] Bussel J. B. . AMG 531, a thrombopoiesis-stimulating protein, for chronic ITP. N. Engl. J. Med. 355, 1672–1681 (2006). 17050891 10.1056/NEJMoa054626

[b10] Portielje J. E., Westendorp R. G., Kluin-Nelemans H. C. & Brand A. Morbidity and mortality in adults with idiopathic thrombocytopenic purpura. Blood 97, 2549–2554 (2001). 11313240 10.1182/blood.v97.9.2549

[b11] Stasi R. & Provan D. Management of immune thrombocytopenic purpura in adults. Mayo Clin. Proc. 79, 504–522 (2004). 15065616 10.4065/79.4.504

[b12] Tomiyama Y. . A lower starting dose of eltrombopag is efficacious in Japanese patients with previously treated chronic immune thrombocytopenia. J. Thromb. Haemost. 10, 799–806 (2012). 10.1111/j.1538-7836.2012.04695.x 22409309

[b13] Cheng G. . Eltrombopag for management of chronic immune thrombocytopenia (RAISE): a 6-month, randomised, phase 3 study. Lancet 377, 393–402 (2011). 20739054 10.1016/S0140-6736(10)60959-2

[b14] Bussel J. B. . Effect of eltrombopag on platelet counts and bleeding during treatment of chronic idiopathic thrombocytopenic purpura: a randomised, double-blind, placebo-controlled trial. Lancet 373, 641–648 (2009). 19231632 10.1016/S0140-6736(09)60402-5

[b15] Kuter D. J. . Efficacy of romiplostim in patients with chronic immune thrombocytopenic purpura: a double-blind randomised controlled trial. Lancet 371, 395–403 (2008). 18242413 10.1016/S0140-6736(08)60203-2

[b16] Tarantino M. D. . Romiplostim in children with immune thrombocytopenia: a phase 3, randomised, double-blind, placebo-controlled study. Lancet 388, 45–54 (2016). 27103127 10.1016/S0140-6736(16)00279-8

[b17] Kuter D. J. . Long-term treatment with romiplostim in patients with chronic immune thrombocytopenia: safety and efficacy. Br. J. Haematol. 161, 411–423 (2013). 23432528 10.1111/bjh.12260

[b18] Tarantino M. D., Fogarty P., Mayer B., Vasey S. Y. & Brainsky A. Efficacy of eltrombopag in management of bleeding symptoms associated with chronic immune thrombocytopenia. Blood Coagul. Fibrinolysis 24, 284–296 (2013). 23492914 10.1097/MBC.0b013e32835fac99

[b19] Grainger J. D. . Eltrombopag for children with chronic immune thrombocytopenia (PETIT2): a randomised, multicentre, placebo-controlled trial. Lancet 386, 1649–1658 (2015). 26231455 10.1016/S0140-6736(15)61107-2

[b20] Shirasugi Y. . Romiplostim for the treatment of chronic immune thrombocytopenia in adult Japanese patients: a double-blind, randomized Phase III clinical trial. Int. J. Hematol. 94, 71–80 (2011). 21706145 10.1007/s12185-011-0886-8

[b21] Kuter D. J. . Romiplostim or standard of care in patients with immune thrombocytopenia. N. Engl. J. Med. 363, 1889–1899 (2010). 21067381 10.1056/NEJMoa1002625

[b22] Elalfy M. S., Abdelmaksoud A. A. & Eltonbary K. Y. Romiplostim in children with chronic refractory ITP: randomized placebo controlled study. Ann. Hematol. 90, 1341–1344 (2011). 21318572 10.1007/s00277-011-1172-9

[b23] Bussel J. B. . Eltrombopag for the treatment of children with persistent and chronic immune thrombocytopenia (PETIT): a randomised, multicentre, placebo-controlled study. Lancet Haematol. 2, doi: 10.1016/s2352-3026(15)00114-3 (2015). 26688484

[b24] Bussel J. B. . Eltrombopag for the treatment of chronic idiopathic thrombocytopenic purpura. N. Engl. J. Med. 357, 2237–2247 (2007). 18046028 10.1056/NEJMoa073275

[b25] Bussel J. B. . A randomized, double-blind study of romiplostim to determine its safety and efficacy in children with immune thrombocytopenia. Blood 118, 28–36 (2011). 21502541 10.1182/blood-2010-10-313908

[b26] Kuter D. J. . Evaluation of bone marrow reticulin formation in chronic immune thrombocytopenia patients treated with romiplostim. Blood 114, 3748–3756 (2009). 19671919 10.1182/blood-2009-05-224766

[b27] Saleh M. N. . Update on the safety and efficacy of EXTENDed treatment with eltrombopag (EPAG) in adults with chronic immune thrombocytopenia (ITP). Blood 122, 2315–2315 (2013).

[b28] Zeng Y., Duan X., Xu J. & Ni X. TPO receptor agonist for chronic idiopathic thrombocytopenic purpura. Cochrane Database Syst. Rev., doi: 10.1002/14651858.CD008235.pub2 (2011). PMC10445271 21735426

[b29] Stasi R. . Evaluation of bleeding-related episodes in patients with immune thrombocytopenia (ITP) receiving romiplostim or medical standard of care. Int. J. Hematol. 96, 26–33 (2012). 22562409 10.1007/s12185-012-1088-8

[b30] Zhou H. . A multicenter randomized open-label study of rituximab plus rhTPO vs rituximab in corticosteroid-resistant or relapsed ITP. Blood 125, 1541–1547 (2015). 25575541 10.1182/blood-2014-06-581868 PMC4351949

[b31] Wang S. . A multicenter randomized controlled trial of recombinant human thrombopoietin treatment in patients with primary immune thrombocytopenia. Int. J. Hematol. 96, 222–228 (2012). 22753022 10.1007/s12185-012-1124-8

[b32] Gernsheimer T. B. . Evaluation of bleeding and thrombotic events during long-term use of romiplostim in patients with chronic immune thrombocytopenia (ITP). J. Thromb. Haemost. 8, 1372–1382 (2010). 20230419 10.1111/j.1538-7836.2010.03830.x

[b33] Buchanan G. R. & Adix L. Grading of hemorrhage in children with idiopathic thrombocytopenic purpura. J. Pediatr. 141, 683–688 (2002). 12410198 10.1067/mpd.2002.128547

[b34] Higgins J. P. . The Cochrane Collaboration’s tool for assessing risk of bias in randomised trials. BMJ 343, doi: 10.1136/bmj.d5928 (2011). PMC3196245 22008217

[b35] Higgins J. P. & Thompson S. G. Quantifying heterogeneity in a meta-analysis. Stat. Med. 21, 1539–1558 (2002). 12111919 10.1002/sim.1186

