# Supplementary material for: Efficacy and safety of thrombopoietin receptor agonists in patients with primary immune thrombocytopenia: A systematic review and meta-analysis
Source: Sci Rep. 2016 Dec 19;6:39003. doi: 10.1038/srep39003 (PMC5171907; doi:10.1038/srep39003)
Supplement: Supplementary Information [file srep39003-s1.pdf]

Efficacy and safety of thrombopoietin receptor agonists in patients with primary immune thrombocytopenia: A systematic review and meta-analysis

Li Wang, Zhe Gao, Xiao-ping Chen, Hai-yan Zhang, Nan Yang, Fei-yan Wang, Li-xun Guan, Zhen-yang Gu, Sha-sha Zhao, Lan Luo, Hua-ping Wei, Chun-ji Gao

Table S1. Characteristics of the excluded full-text studies.

Study	Reason for exclusion
Tarantino *et al.*[1]	Retrospective analysis of pooled clinical trials
Michel *et al.*[2]	Retrospective analysis of pooled clinical trials
Stasi *et al.*[3]	No outcomes of interest (outcome: bleeding-related episodes)
Kuter *et al.*[4]	No outcomes of interest (outcome: health-related QOL)
Klaassen *et al.*[5]	No outcomes of interest (outcomes: health-related QOL and parental burden)
Signorovitch *et al.*[6]	No outcomes of interest (outcome: QOL) and duplicated data
Sanz *et al.*[7]	No outcomes of interest (outcome: QOL) and pooled data
Mathias *et al.*[8]	No outcomes of interest (outcomes: child health-related QOL and parental burden)
Mathias *et al.*[9]	No outcomes of interest (outcome: QOL)
Iskedjian *et al.*[10]	No outcomes of interest (outcome: QOL)
Mathias *et al.*[11]	No outcomes of interest (outcome: QOL)
George *et al.*[12]	No outcomes of interest (outcome: QOL)
Kuter *et al.*[13]	Not a RCT
Mazza *et al.*[14]	Not a RCT and retrospectively compared romiplostim and eltrombopag
Haselboeck *et al.*[15]	Not a RCT
Kuter *et al.*[16]	Extension study (single arm)
Cui *et al.*[17]	Other comparison (rhTPO plus cyclosporine A vs. rhTPO alone)
Gu *et al.*[18]	Other language (Chinese, rhTPO plus glucocorticoid vs. glucocorticoid alone)
Zhou *et al.*[19]	Other TPO-RA (rhTPO + rituximab vs. rituximab)
Wang *et al.*[20]	Other TPO-RA (rhTPO + danazol vs. danazol)
Bussel *et al.*[21]	Other TPO-RA (avatrombopag)

Abbreviations: QOL: quality of life; RCT: randomized controlled trial; rhTPO: recombinant human thrombopoietin; TPO-RA: thrombopoietin receptor agonist.

References

1. Tarantino, M. D., Fogarty, P., Mayer, B., Vasey, S. Y. & Brainsky, A. Efficacy of eltrombopag in management of bleeding symptoms associated with chronic immune thrombocytopenia. *Blood Coagul. Fibrinolysis* **24,** 284-296 (2013).
2. Michel, M. *et al.* Efficacy and safety of the thrombopoietin receptor agonist romiplostim in patients aged >/= 65 years with immune thrombocytopenia. *Ann. Hematol.* **94,** 1973-1980 (2015).
3. Stasi, R. *et al.* Evaluation of bleeding-related episodes in patients with immune thrombocytopenia (ITP) receiving romiplostim or medical standard of care. *Int. J. Hematol.* **96,** 26-33 (2012).
4. Kuter, D. J. *et al.* Health-related quality of life in nonsplenectomized immune thrombocytopenia patients receiving romiplostim or medical standard of care. *Am. J. Hematol.* **87,** 558-561 (2012).
5. Klaassen, R. J. *et al.* Pilot study of the effect of romiplostim on child health-related quality of life (HRQoL) and parental burden in immune thrombocytopenia (ITP). *Pediatr. Blood Cancer* **58,** 395-398 (2012).
6. Signorovitch, J., Brainsky, A. & Grotzinger, K. M. Validation of the FACIT-fatigue subscale, selected items from FACT-thrombocytopenia, and the SF-36v2 in patients with chronic immune thrombocytopenia. *Qual. Life Res.* **20,** 1737-1744 (2011).
7. Sanz, M. A., Aledort, L., Mathias, S. D., Wang, X. & Isitt, J. J. Analysis of EQ-5D scores from two phase 3 clinical trials of romiplostim in the treatment of immune thrombocytopenia (ITP). *Value Health* **14,** 90-96 (2011).
8. Mathias, S. D. *et al.* A phase 3, randomized, double-blind, placebo-controlled study to

determine the effect of romiplostim on health-related quality of life in children with primary immune thrombocytopenia and associated burden in their parents. *Pediatr. Blood Cancer* **63,** 1232-1237 (2016).

9. Mathias, S. D. *et al.* Evaluating clinically meaningful change on the ITP-PAQ: preliminary estimates of minimal important differences. *Curr. Med. Res. Opin.* **25,** 375-383 (2009).

10. Iskedjian, M. *et al.* Elicitation of utility scores in Canada for immune thrombocytopenia treated with romiplostim or watch and rescue. *J. Med. Econ.* **15,** 313-331 (2012).

11. Mathias, S. D. *et al.* A disease-specific measure of health-related quality of life in adults with chronic immune thrombocytopenic purpura: Psychometric testing in an open-label clinical trial. *Clin. Ther.* **29,** 950-962 (2007).

12. George, J. N. *et al.* Improved quality of life for romiplostim-treated patients with chronic immune thrombocytopenic purpura: results from two randomized, placebo-controlled trials. *Br. J. Haematol.* **144,** 409-415 (2009).

13. Kuter, D. J. *et al.* Treatment patterns and clinical outcomes in patients with chronic immune thrombocytopenia (ITP) switched to eltrombopag or romiplostim. *Int. J. Hematol.* **101,** 255-263 (2015).

14. Mazza, P. *et al.* The use of thrombopoietin-receptor agonists (TPO-RAs) in immune thrombocytopenia (ITP): a “real life” retrospective multicenter experience of the Rete Ematologica Pugliese (REP). *Ann. Hematol.* **95,** 239-244 (2016).

15. Haselboeck, J., Kaider, A., Pabinger, I. & Panzer, S. Function of eltrombopag-induced platelets compared to platelets from control patients with immune thrombocytopenia. *Thromb. Haemost.* **109,** 676-683 (2013).

16. Kuter, D. J. *et al.* Long-term treatment with romiplostim in patients with chronic immune thrombocytopenia: safety and efficacy. *Br. J. Haematol.* **161,** 411-423 (2013).

17. Cui, Z. G. *et al.* Recombinant human thrombopoietin in combination with cyclosporin A as a novel therapy in corticosteroid-resistant primary immune thrombocytopenia. *Chin. Med. J. (Engl.)* **126,** 4145-4148 (2013).

18. Gu, S. Y. *et al.* [A clinical comparative study on treatment of severe newly diagnosed immune thrombocytopenia by recombinant human thrombopoietin combined with glucocorticoid]. *Zhonghua Xue Ye Xue Za Zhi* **34,** 883-886 (2013).

19. Zhou, H. *et al.* A multicenter randomized open-label study of rituximab plus rhTPO vs rituximab in corticosteroid-resistant or relapsed ITP. *Blood* **125,** 1541-1547 (2015).

20. Wang, S. *et al.* A multicenter randomized controlled trial of recombinant human thrombopoietin treatment in patients with primary immune thrombocytopenia. *Int. J. Hematol.* **96,** 222-228 (2012).

21. Bussel, J. B. *et al.* A randomized trial of avatrombopag, an investigational thrombopoietin-receptor agonist, in persistent and chronic immune thrombocytopenia. *Blood* **123,** 3887-3894 (2014).

Table S2. PubMed search strategy (from inception to May 22, 2016).

No.	Query results	Results
#12	#4 AND #8 AND #11	186
#11	#9 OR #10	1040321
#10	Search "Randomized Controlled Trials as Topic"[Mesh] OR "Randomized Controlled Trial" [Publication Type]	509044
#9	Search random*	1040002
#8	#5 OR #6 OR #7	7249
#7	Search ((TPO) OR thrombopoietin) OR ("Thrombopoietin"[Mesh] OR "thrombopoietin mimetic peptide" [Supplementary Concept] OR "MPL protein, human" [Supplementary Concept] OR "Receptors, Thrombopoietin"[Mesh])	7112
#6	Search ((((eltrombopag) OR Promacta) OR sb 497115) OR sb-497115) OR "eltrombopag" [Supplementary Concept]	448
#5	Search ((((((Romiplostim) OR amg531) OR amg 531) OR amg-531) OR "romiplostim" [Supplementary Concept])) OR Nplate	416
#4	#1 OR #2 OR #3	67074
#3	Search ITP	10655
#2	Search "Thrombocytopenia"[Mesh] OR "Thrombocytopenia, Neonatal Alloimmune"[Mesh] OR "Purpura, Thrombocytopenic, Idiopathic"[Mesh]	41790
#1	Search thrombocytopeni*	61174

Table S3. Embase search strategy (from inception to May 22, 2016).

No.	Query results	Results
#12	#4 AND #8 AND #11	382
#11	#9 OR #10	1249435
#10	'randomized controlled trial'/exp	397390
#9	random*	1249435
#8	#5 OR #6 OR #7	11191
#7	(TPO: ti,ab) OR (thrombopoietin: ti,ab) OR ('thrombopoietin'/exp)	10208
#6	(eltrombopag: ti,ab) OR (Promacta: ti,ab) OR (sb AND 497115: ti,ab) OR (sb 497115: ti,ab) OR ('eltrombopag'/exp)	1280
#5	(Romiplostim: ti,ab) OR (amg531: ti,ab) OR (amg 531: ti,ab) OR (amg AND 531: ti,ab) OR (Nplate: ti,ab) OR ('romiplostim'/exp)	1291
#4	#1 OR #2 OR #3	81139
#3	itp:ab,ti	8568
#2	'idiopathic thrombocytopenic purpura'/exp	12613
#1	thrombocytopeni*:ab,ti	75900

Table S4. CENTRAL search strategy (from inception to May 22, 2016).

No.	Query results	Results
#9	#4 AND #8	228
#8	#5 OR #6 OR #7	351
#7	(thrombopoietin:ti,ab,kw) OR (TPO:ti,ab,kw) OR (MeSH descriptor: [Thrombopoietin] explode all trees)	278
#6	(eltrombopag:ti,ab,kw) OR (Promacta:ti,ab,kw) OR (sb-497115:ti,ab,kw) OR (sb 497115:ti,ab,kw)	98
#5	(Romiplostim:ti,ab,kw) OR (amg531:ti,ab,kw) OR (amg-531:ti,ab,kw) OR (amg 531:ti,ab,kw) OR (Nplate:ti,ab,kw)	78
#4	#1 OR #2 OR #3	5210
#3	MeSH descriptor: [Purpura, Thrombocytopenic, Idiopathic] explode all trees	162
#2	ITP:ti,ab,kw (Word variations have been searched)	283
#1	thrombocytopeni*:ti,ab,kw (Word variations have been searched)	5175

Note:

Cochrane Central Register of Controlled Trials (Issue 4 of 12, April 2016): 209

Cochrane reviews (4)

Other reviews (1)

Technology assessments (12)

Economic evaluations (2)

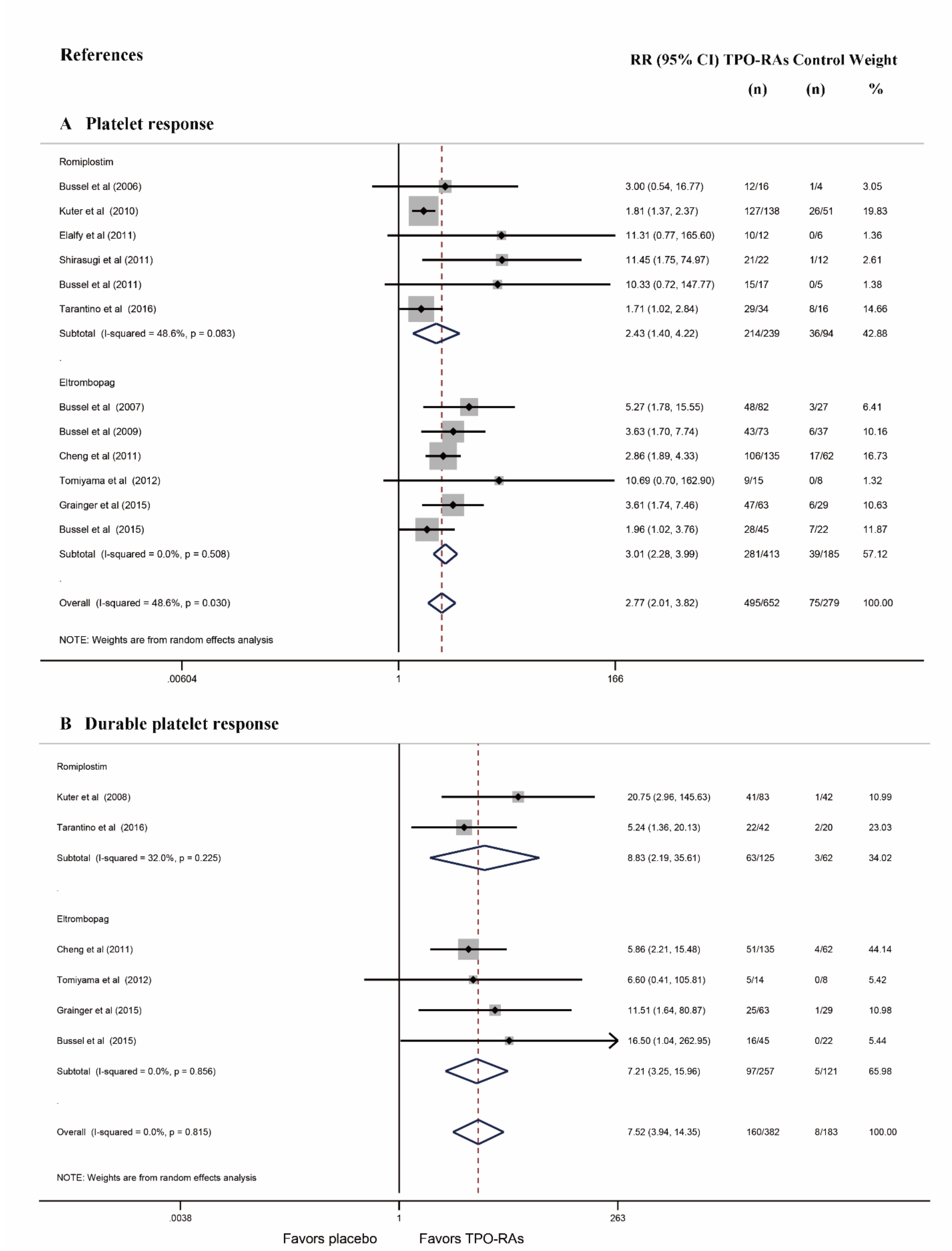

-

Figure S1. Forest plot and meta-analysis of the rates of R and DR. Subgroup meta-analysis based on TPO-RA regimens (romiplostim vs. eltrombopag). TPO-RA: thrombopoietin receptor agonist; RR: risk ratio; CI: confidence interval.

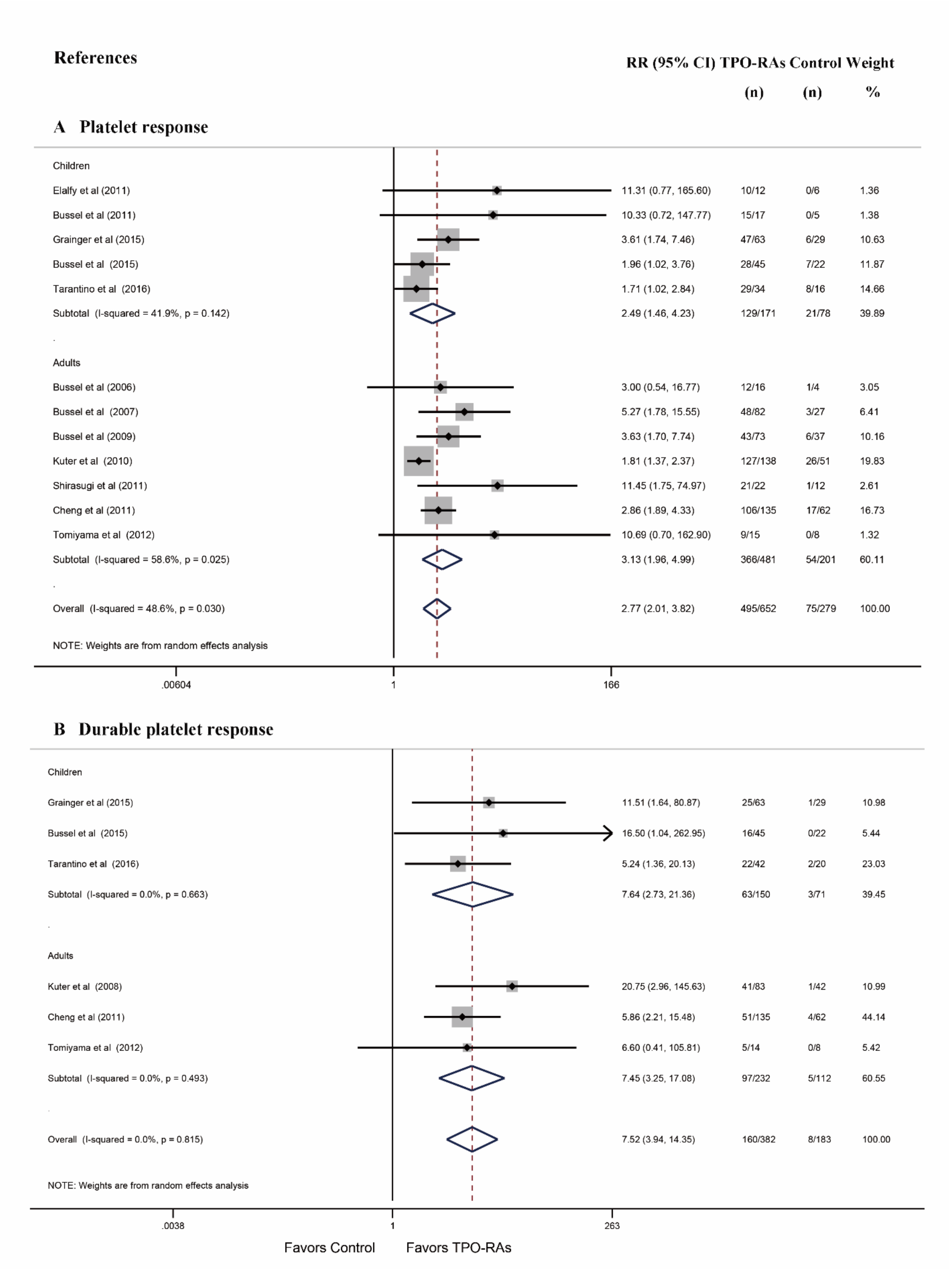

Figure S2. Forest plot and meta-analysis of the rates of R and DR. Subgroup meta-analysis based on patient populations (children vs. adults). TPO-RA: thrombopoietin receptor agonist; RR: risk ratio; CI: confidence interval.

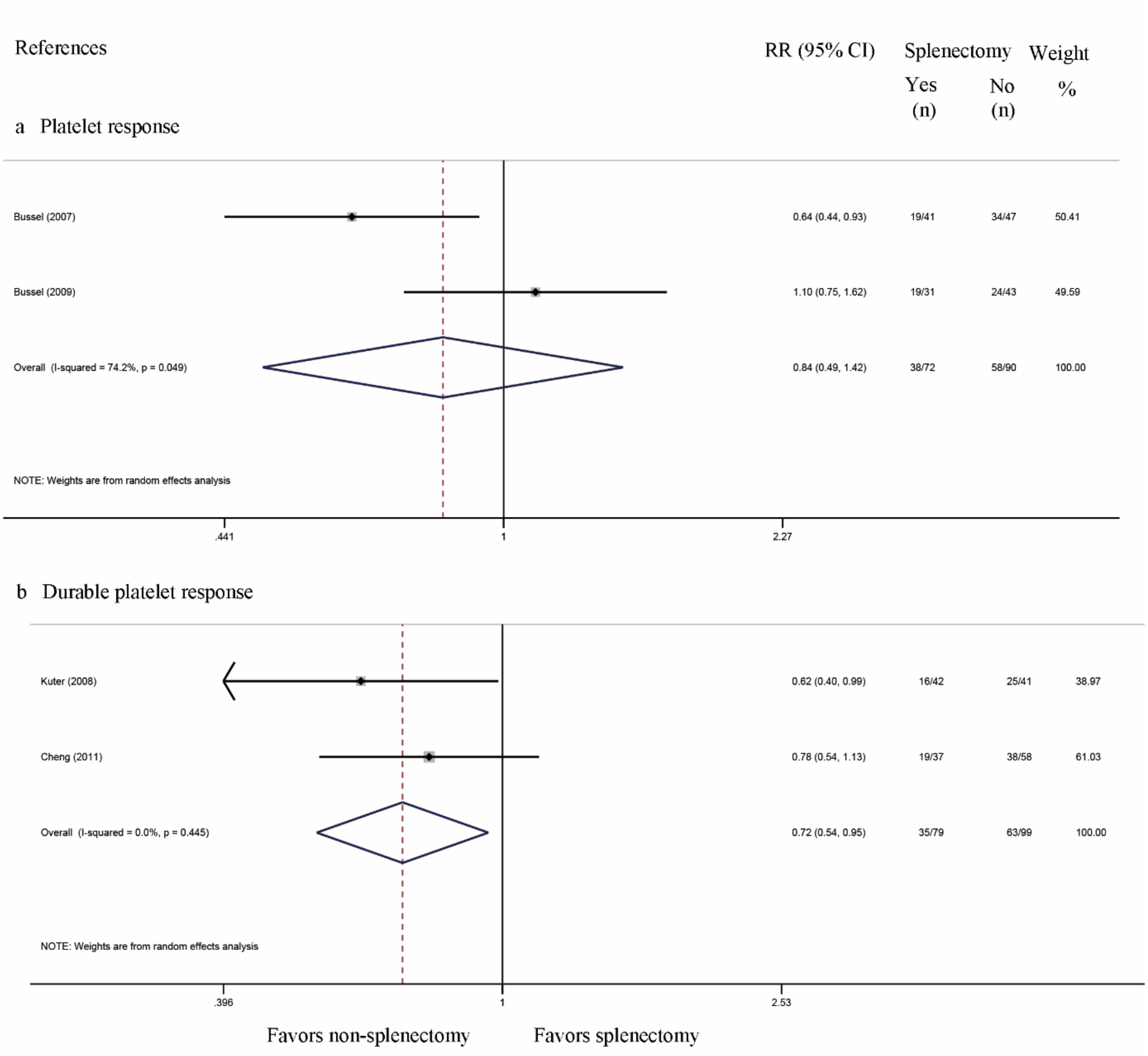

Figure S3. Forest plot and meta-analysis of the rates of R and DR. Subgroup meta-analysis based on splenectomy status (splenectomy vs. non-splenectomy). RR: risk ratio; CI: confidence interval.

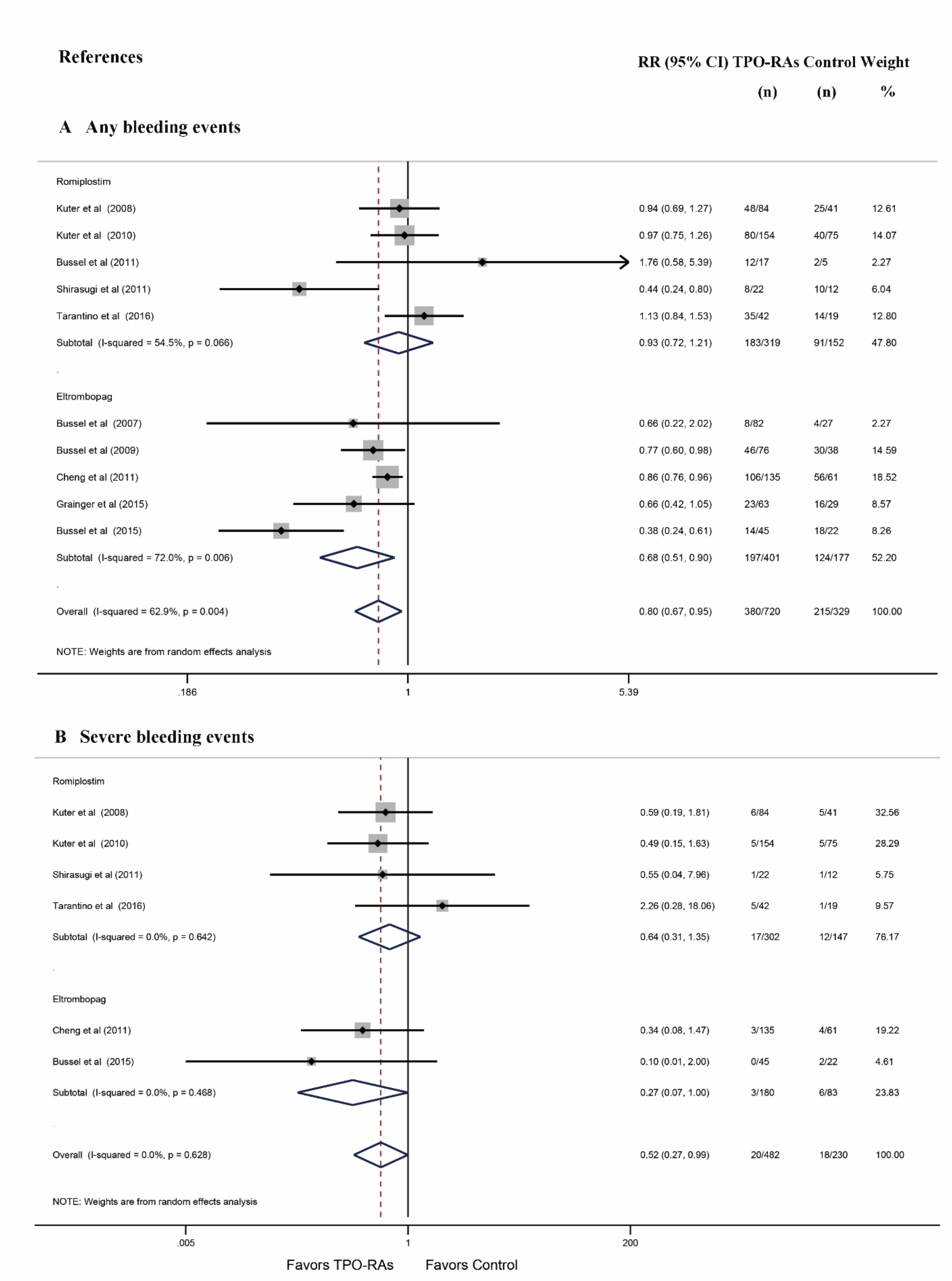

Figure S4. Forest plot and meta-analysis of the incidence of any or severe bleeding events. Subgroup meta-analysis based on TPO-RA regimens (romiplostim vs. eltrombopag). TPO-RA: thrombopoietin receptor agonist; RR: risk ratio; CI: confidence interval.

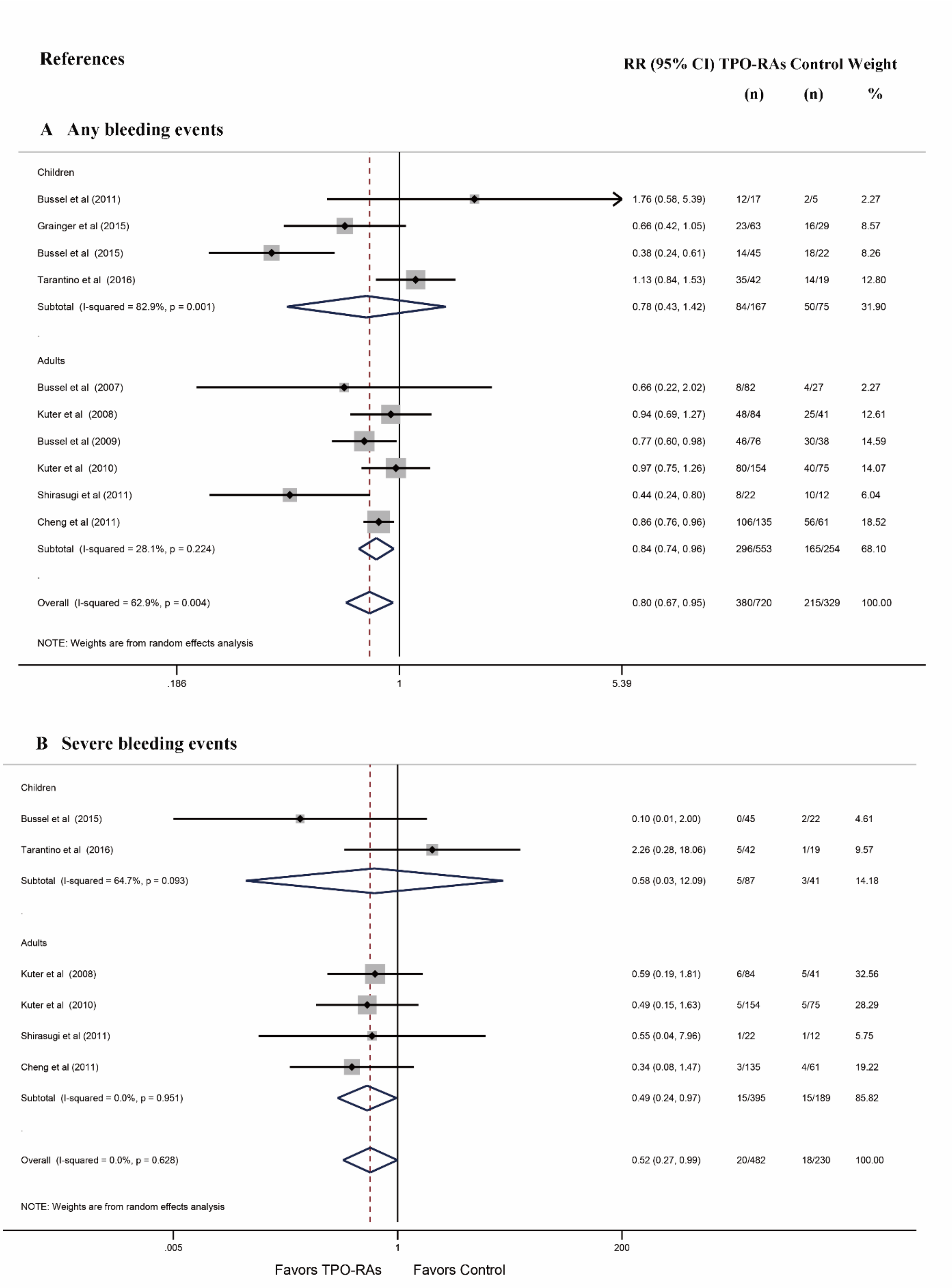

Figure S5. Forest plot and meta-analysis of the incidence of any or severe bleeding events. Subgroup meta-analysis based on patient populations (children vs. adults). TPO-RA: thrombopoietin receptor agonist; RR: risk ratio; CI: confidence interval.

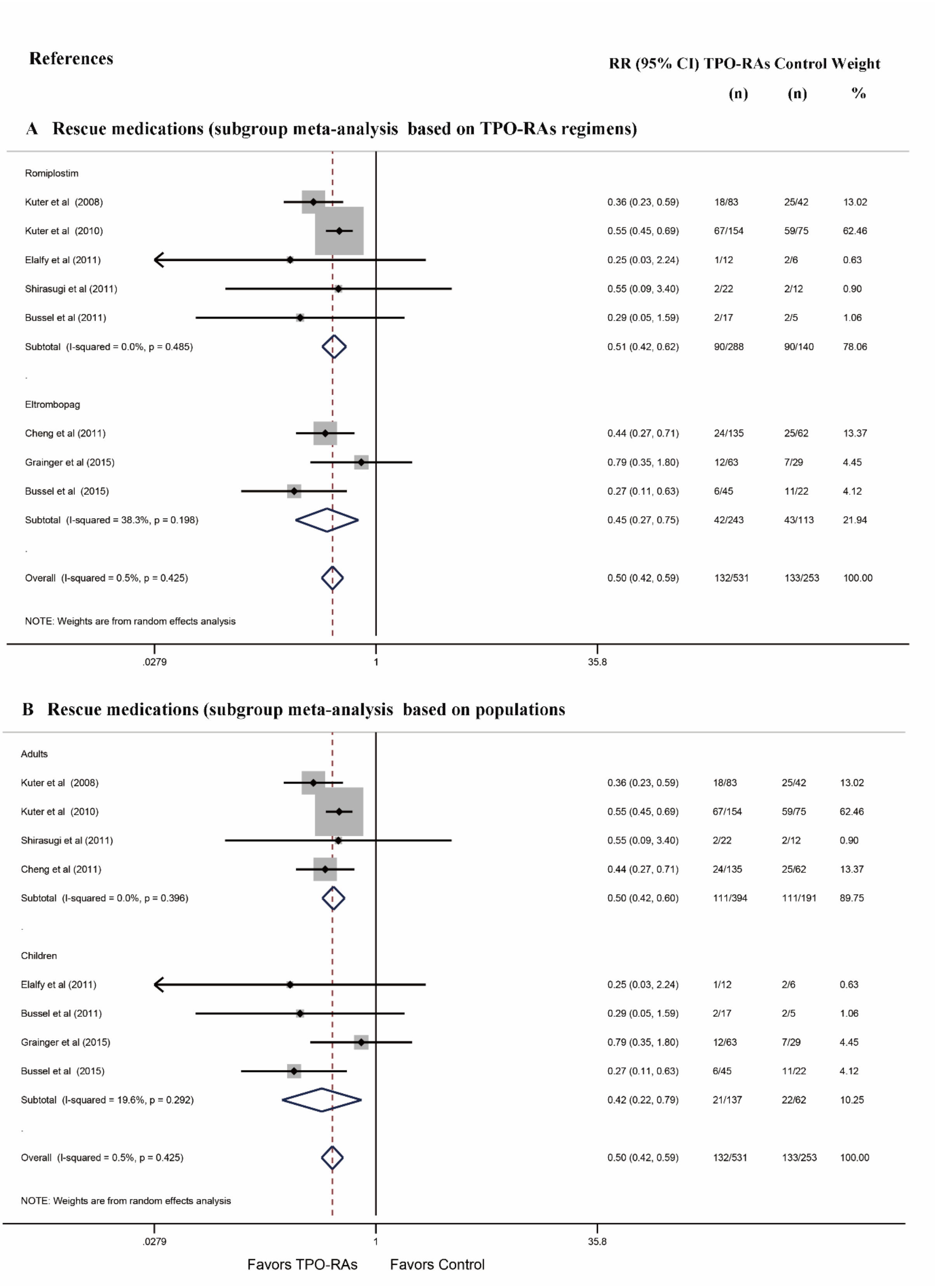

Figure S6. Forest plot and meta-analysis of the need for rescue medications. Subgroup meta-analysis based on TPO-RA regimens (romiplostim vs. eltrombopag) or patient populations (children vs. adults). TPO-RA: thrombopoietin receptor agonist; RR: risk ratio; CI: confidence interval.

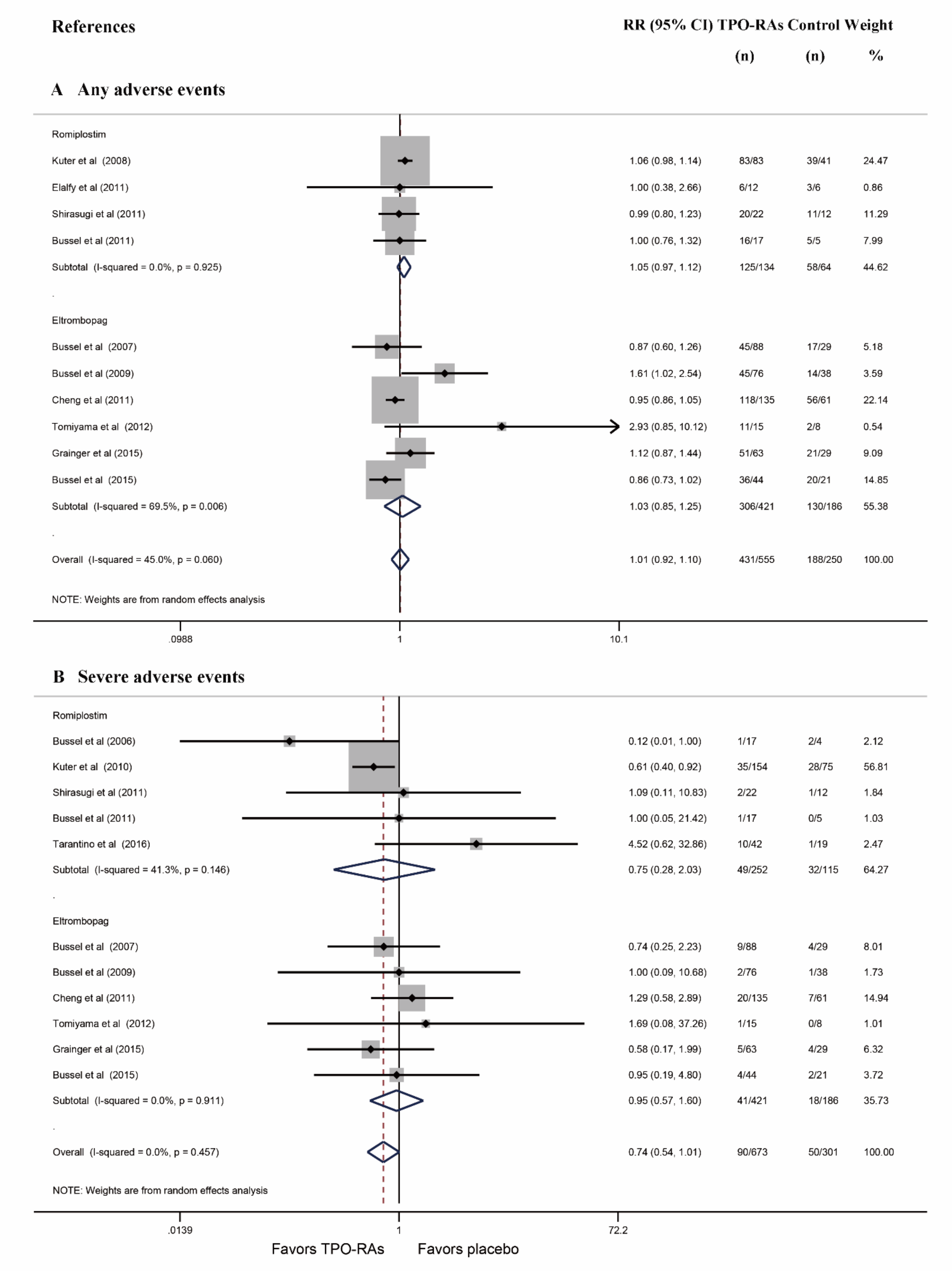

Figure S7. Forest plot and meta-analysis of the incidence of any or severe adverse events. Subgroup meta-analysis based on TPO-RA regimens (romiplostim vs. eltrombopag). TPO-RA: thrombopoietin receptor agonist; RR: risk ratio; CI: confidence interval.

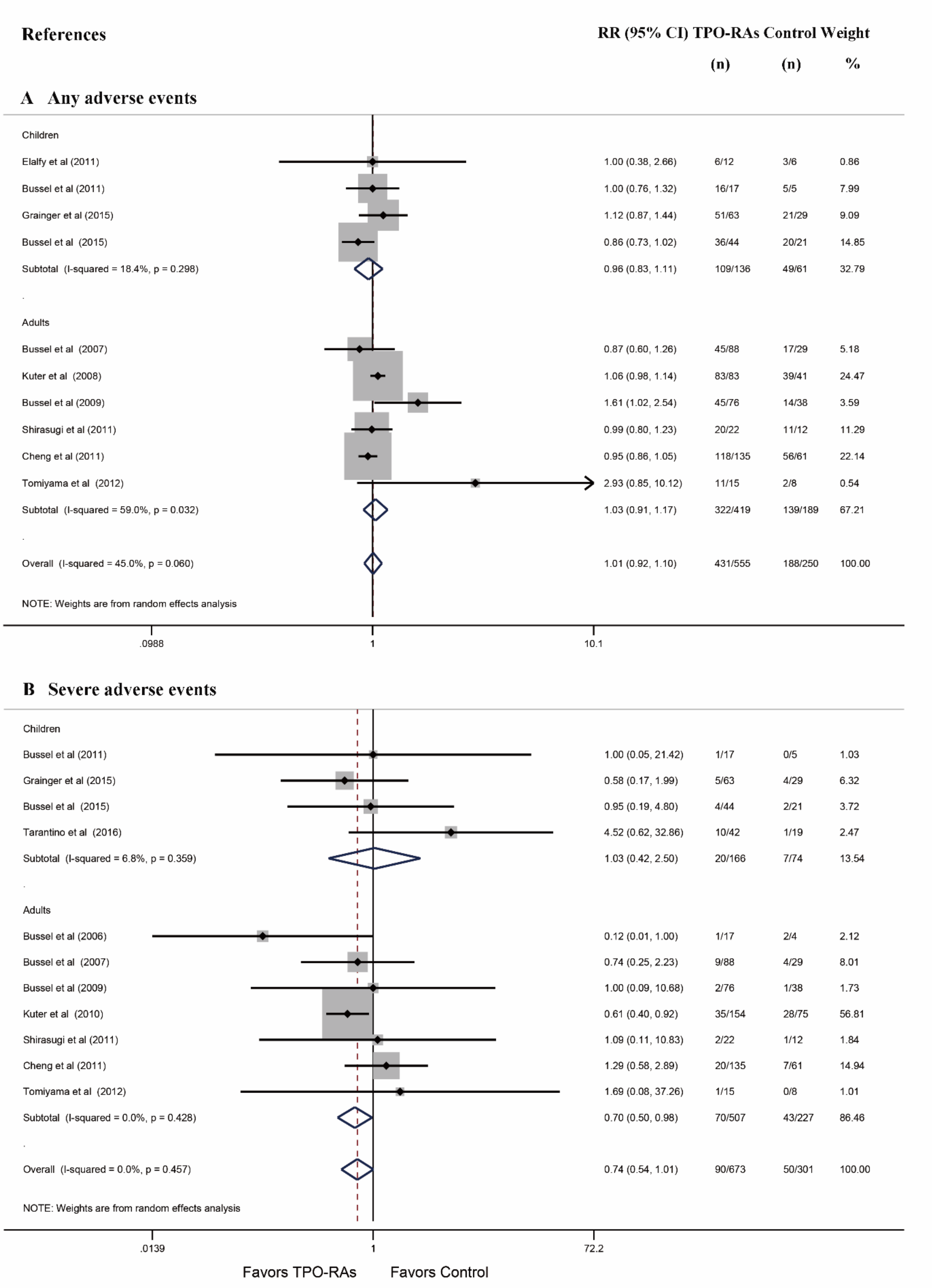

Figure S8. Forest plot and meta-analysis of the incidence of any or severe adverse events. Subgroup meta-analysis based on patient populations (children vs. adults). TPO-RA: thrombopoietin receptor agonist; RR: risk ratio; CI: confidence interval.

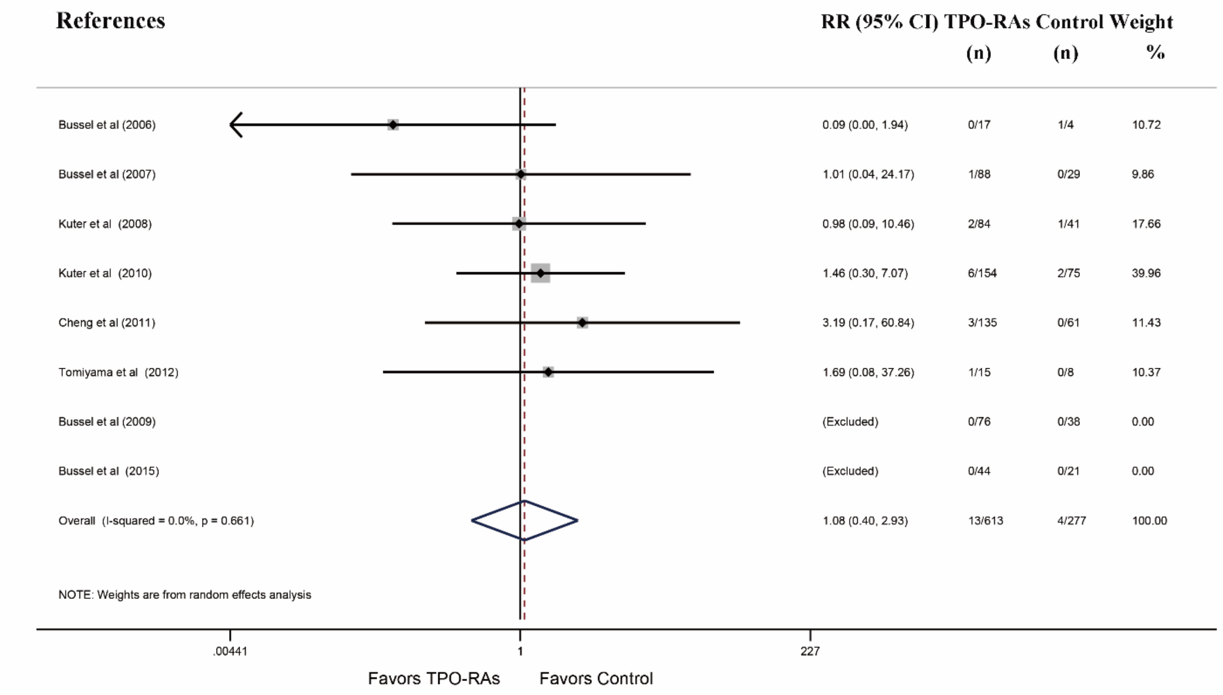

Figure S9. Forest plot and meta-analysis of the incidence of thrombotic events. TPO-RA: thrombopoietin receptor agonist; RR: risk ratio; CI: confidence interval.

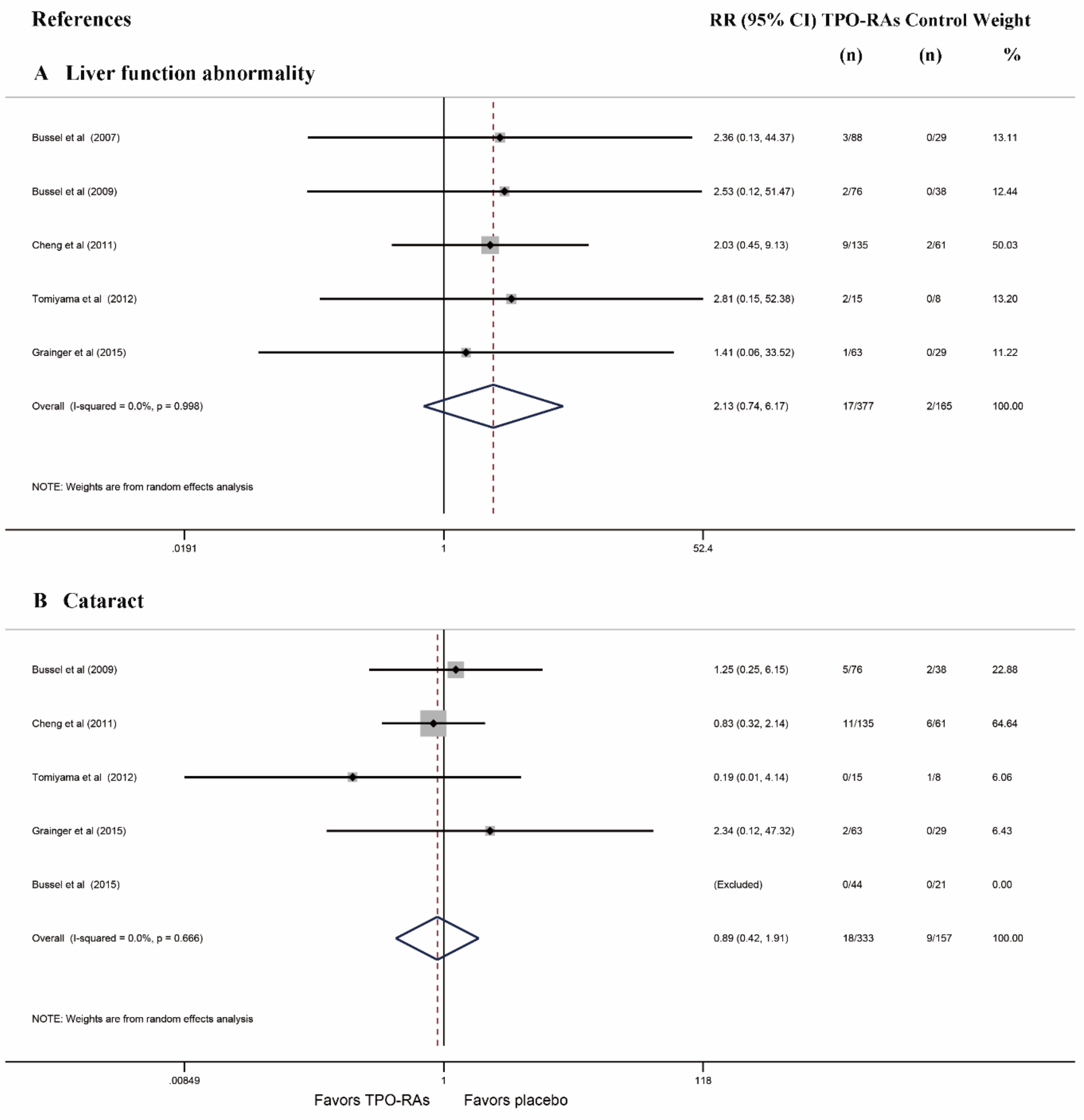

Figure S10. Forest plot and meta-analysis of the incidences of liver function abnormalities and cataracts. TPO-RAs: thrombopoietin receptor agonists; RR: risk ratio; CI: confidence interval.
